# Direct probing of low-energy intra *d*-band transitions in gas-phase cobalt clusters

**DOI:** 10.1038/s42004-024-01206-2

**Published:** 2024-06-04

**Authors:** Kevin A. Kaw, Rick J. Louwerse, Joost M. Bakker, Peter Lievens, Ewald Janssens, Piero Ferrari

**Affiliations:** 1 Quantum Solid-State Physics, Department of Physics and Astronomy, KU Leuven, Celestijnenlaan 200d, 3001 Leuven, Belgium; 2 Radboud University, Institute for Molecules and Materials, HFML-FELIX, 6525 Nijmegen, ED Netherlands

**Keywords:** Excited states, Infrared spectroscopy, Infrared spectroscopy

## Abstract

The interplay between constituent localized and itinerant electrons of metal clusters defines their physical and chemical properties. In turn, the electronic and geometrical structures are strongly entwined and exhibit strong size-dependent variations. Current understanding of low-energy excited states of metal clusters relies on stand-alone theoretical investigations and few comparisons with measured properties, since direct identification of low-lying states is lacking hitherto. Here, we report on the measurement of low-lying electronic transitions in cationic cobalt clusters using infrared photofragmentation spectroscopy. Broad and size-dependent absorption features were observed within 0.056 – 0.446 eV, well above the energies of the sharp absorption bands caused by cluster vibrations. Complementary time-dependent density functional theory calculations reproduce the main observed absorption features, providing direct evidence that they correspond to transitions between electronic states of mainly *d*-character, arising from the open *d*-shells of the Co atoms and the high spin multiplicity of the clusters.

## Abstract (web summary)

Large classes of metal clusters are expected to have dipole-allowed electronic transitions in the near-infrared, but precise experimental characterization of such low-lying states is lacking. Here, the authors probe cationic cobalt clusters of 4 to 15 atoms using IR photofragmentation spectroscopy with krypton as a messenger atom to show that low-lying electronic excited states are responsible for the size-dependent radiative decay of these highly excited clusters.

## Introduction

Most fundamental properties of metal clusters are linked to their electronic structure, in particular at low excitation energies. For example, the energy difference between the highest occupied and lowest unoccupied molecular orbitals (HOMO and LUMO) plays a fundamental role in cluster reactivity^1,2^, whereas open electronic shells allow for unusually high magnetic moments in comparison to the bulk^3,4^. For basic size-dependent properties such as the relative stability of clusters, the electronic structure at low excitation energies is crucial, with particularly stable magic clusters having large HOMO-LUMO gaps and closed electronic configurations^5,6^. Recent studies have attributed a decisive role to low-lying electronic states in the efficiency of recurrent fluorescence (RF), a process in which energy excess is dissipated via photon emission from excited electronic states thermally populated through a coupling to vibrationally excited states. RF is often neglected as a fast internal energy dissipation mechanism^7,8^. RF rates as high as 10^6^ s^−1^ have been quantified in metal clusters, which can only be rationalized by the involvement of electronic transitions well below 1 eV^9^. Therefore, it is hypothesized that large classes of clusters have dipole-allowed electronic transitions in the near infrared, but the identification and precise characterization of such low-lying states is lacking.

In our study, we focus on the density of states (DOS) at low excitation energies of cationic cobalt clusters, Co_*n*_^+^ (*n* = 4–15), for which previous studies have hypothesized the existence of low-lying electronic states. Recently, Peeters et al. determined RF rates of laser excited Co_*n*_^+^ (*n* = 5–23) clusters, revealing high rates in the 10^6^ s^−1^ range^10^. Cunningham et al. employed infrared spectroscopy to investigate the catalytic decomposition of nitrous oxide on Co_*n*_^+^, proposing an involvement of low-lying excited states in the binding of N_2_O^11^. Jalink et al. used UV photoionization spectroscopy to show the IR-induced thermal repopulation of electronically excited states in Co_*n*_ (*n* = 9, 10, 13), with energies of typically 0.1 eV^12^. Minemoto et al. performed photodissociation spectroscopy measurements on Co_*n*_^+^Ar (*n* = 3–5) clusters for energies above 0.7 eV, finding absorption features above 1 eV^13^. Zamudio-Bayer et al. employed x-ray magnetic circular dichroism (XMCD) spectroscopy measurements to attribute high-spin multiplicities in Co_*n*_^+^ to a dense DOS of singly occupied molecular orbitals around the HOMO^14,15^. Hence, evidence points to the presence of these low-lying electronic states in cobalt clusters, which can be relevant in other transition metal clusters too, highlighting the crucial need for direct spectroscopic characterization.

The most direct experimental method to access the electronic structure of clusters is optical absorption spectroscopy^16^, where electronic transitions are determined from the energies at which clusters resonantly absorb light^17^. In this article, we report on the direct experimental identification of low-lying electronic transitions in cobalt cationic clusters, detected via near-IR photofragmentation spectroscopy of Kr-tagged Co_*n*_^+^ (*n* = 4–15) clusters, using the laser light of an infrared free-electron laser. The experimental work is complemented by density functional theory (DFT) and time-dependent DFT (TD-DFT) calculations, using an all-electron basis set and directly treating relativistic effects, in conjunction with an exchange-correlation functional proved to behave well for transition metal clusters.

## Results and discussion

### Mid-IR photofragmentation spectroscopy of cobalt clusters

Typically, gas-phase optical absorption experiments employ photofragmentation, where the elimination of a weakly bound messenger signals resonant optical absorption. With this technique, however, sensitivity at low excitation energies is reduced if the messenger-cluster binding energy is similar to or higher than the photon energy, impeding a detailed characterization of low-lying electronic states. Recent photofragmentation experiments have shown what appears to be the signature of a low-lying electronic transition for Au_10_Ar^+^, reflected in a broad absorption feature in the mid-infrared range^18^, similar to an onset reported for Ta_4_^+^^19^.

**Fig. 1:**
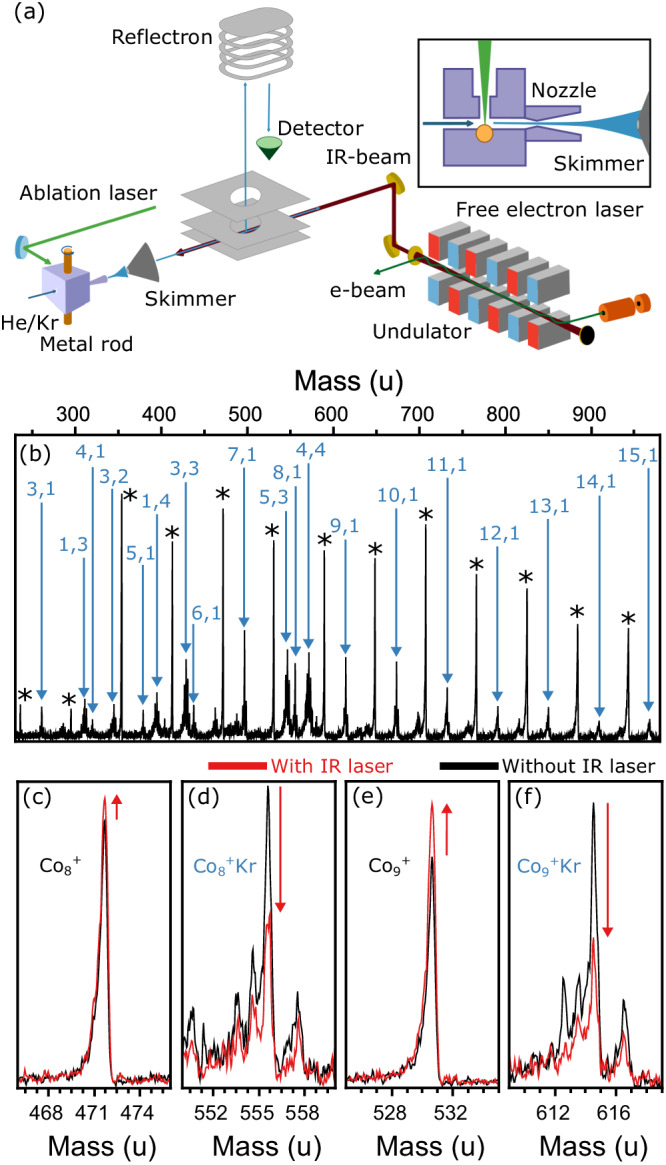
Summary of the experimental procedure. **a** Scheme of the laser ablation cluster source and time-of-flight mass spectrometer connected to the beam line of the FELIX free-electron laser (more details in the Methods section). **b** Mass spectrum of Co_*n*_^+^Kr_*m*_ clusters, with peaks labeled as *n*, *m*. Bare Co_*n*_^+^ clusters (*n* = 3–15) are marked by asterisks. **c**–**f** Effect of IR irradiation at 0.28 eV (2260 cm^−1^) on the mass distribution (black without and red with IR light).

In this work, experiments are performed in a molecular beam instrument coupled to the FELIX free-electron laser in Nijmegen, The Netherlands, as sketched in Fig. 1a. A distribution of Co_*n*_^+^ and Co_*n*_^+^Kr_*m*_ (*n* ≤ 15) complexes is formed with Kr acting as messenger, which is analyzed by time-of-flight mass spectrometry. A typical mass spectrum is shown in Fig. 1b. Several Kr atoms are attached per cluster for *n* ≤ 5, while clusters with *n* ≥ 6 typically adsorb only a single Kr atom. A similar trend was observed for Ar complexes^20^.

To record infrared spectra, the cluster distribution is irradiated by a counterpropagating IR laser beam from the free-electron laser. Absorption spectra were constructed by comparing the cluster intensities in the mass spectra with and without IR light. Resonant photoexcitation of Co_*n*_^+^Kr_*m*_ leads to the loss of one (or more) Kr atoms, and consequentially to a reduction of the Co_*n*_^+^Kr_*m*_ signal and a corresponding increase for Co_*n*_^+^Kr_*m*−1_. This behavior is illustrated in Fig. 1c–f (*n* = 8 and 9). Crucial for the current experiment is that even if the covered photon energy is lower than the Kr binding energy, the use of FELIX allows for resonant multiple photon absorption, sufficient to surpass the messenger desorption threshold. Therefore, the experiment is sensitive to low-energy excitations.

**Fig. 2:**
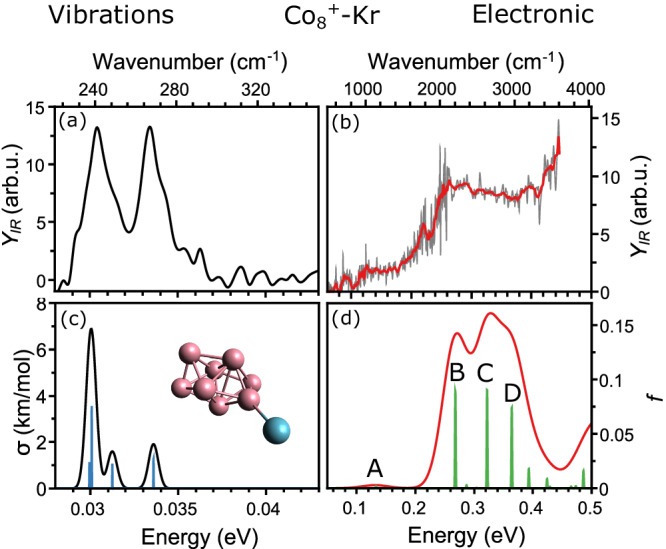
Photofragmentation spectra of Co_8_^+^Kr. **a**, **b** Spectrum of Co_8_^+^Kr in the far- (**a**) and mid-infrared (**b** 10-point running average in red). The top axis presents the spectral range in wavenumber, the bottom axis in energy. FELIX power was ~10 times larger below 2000 cm^−1^, resulting in a higher signal-to-noise ratio. **c**, **d** DFT and TD-DFT calculated spectra, convolved with Gaussian line shape functions to facilitate comparison with the experiment. The labels A, B, C and D highlight transitions discussed in the text.

The IR absorption at frequency *ω* is quantified by the depletion yield *Y*_IR_(*ω*), defined as

1 $${Y}_{{{{{{\rm{IR}}}}}}}(\omega )=-\,{{{{{\mathrm{ln}}}}}\left(\frac{I(\omega )}{{I}_{0}(\omega )}\right)}/{E}_{{{{{{\rm{IR}}}}}}}(\omega ),$$

with *I*(ω) and *I*_0_(ω) the intensities in the mass channels of a cluster with and without IR light excitation, respectively, and *E*_IR_(ω) the laser pulse energy ^21^. Figure 2a, b shows the absorption spectrum of Co_8_^+^Kr. For simplicity, the full spectrum is separated into two frames: the far-infrared region in (a), and the mid-infrared range in (b). The far-infrared range shows two clear absorption bands that can readily be attributed to vibrational modes of Co_8_^+^Kr, at frequencies consistent with those previously detected in Co_8_^+^Ar complexes^20^. Small shifts in band frequencies between Co_8_^+^Ar and Co_8_^+^Kr are observed, which can be attributed to the different tagging element (see Figure S1). More interestingly, and not previously observed, is the broad absorption feature in the mid-infrared range, starting roughly at 0.1 eV and showing an underlying structure composed of local maxima at ~0.12, 0.25 and 0.31 eV. Above 0.4 eV, there is further signal increase, with a maximum not found within the accessible spectral range. Since the exact photon flux is unknown, only relative cross sections can be inferred.

### Assignment of low-energy transitions and states

The ground state geometry of selected cationic cobalt clusters tagged with a Kr atom were calculated using DFT at the PBE/Def2-TZVPP level of theory. Relativistic effects are considered implicitly by using an all-electron approach and the zeroth order regular approximation (ZORA). Previous far-infrared spectroscopy experiments have assigned the geometries of Co_*n*_^+^ clusters for *n* ≤ 8, which we adopt here for the computations^20^. Figure 2c depicts the calculated harmonic vibrational transitions of Co_8_^+^Kr, convolved with Gaussian line shape functions (10 cm^−1^ FWHM). The cluster geometry is shown as inset. A comparison of the frequency calculations and the absorption spectrum in the far-infrared (Fig. 2a) reveals good agreement, with calculated bands at 0.030 and 0.034 eV well reproduced, including a shoulder at ~0.032 eV, even if the intensity of the high frequency band is somewhat lower than observed. All vibrations are predicted below 0.037 eV (300 cm^−1^). Therefore, the broad absorption features seen in the mid-infrared region of the spectrum (b) cannot be attributed to vibrations.

To explore the premise that the mid-infrared experimental features correspond to electronic excitations, results from the TD-DFT calculations for Co_8_^+^Kr are presented in Fig. 2d. We found several electronic transitions of significant oscillator strength below 0.5 eV. The lowest frequency transition, corresponding to the HOMO → LUMO excitation, is found at 0.13 eV (labeled A in the figure), with more intense transitions occurring at 0.27, 0.32 and 0.36 eV (B, C, D). A convolution with Gaussian functions with a 0.05 eV width shows a fairly good agreement with the experimental results. Therefore, the computations strongly suggest that the structure observed in the mid-infrared spectrum indeed corresponds to electronic transitions. All clusters in the *n* = 4–15 size range show similar bands that can be attributed to low-lying electronic states, as described in Supplementary Note 1 and shown in Figures S2–S7. Interestingly, without direct inclusion of relativistic effects, the lowest predicted transitions for Co_8_^+^Kr are found higher in energy, close to 0.5 eV (Figure S8). We acknowledge that the use of TD-DFT to describe low-lying electronic excitations in Co_*n*_^+^ clusters can be an oversimplification of the possible multiconfigurational character of their wavefunctions^22^, but still, our treatment correctly predicts electronic transitions at the energies seen experimentally. Despite the simplification TD-DFT can represent, our applied computational method seems to capture the main electronic information, bearing in mind that perfect quantitative reproduction of the experimental data cannot be expected.

Interestingly, the mechanism of photoexcitation of electronic states resulting in the loss of Kr must involve a coupling between electronic and vibrational states (internal conversion), with efficient intramolecular vibrational redistribution (IVR) to channel the energy into the Co_*n*_^+^-Kr coordinate. The observation of vibrational modes at photon energies well below the Co_*n*_^+^-Kr binding energy is testament that IVR is efficient. This process is particularly likely given the peculiar FELIX pulse structure of 10 µs pulse trains of picosecond pulses, giving time for IVR in between pulses^23^. Whether the current electronic transitions are revealed through single or multiple photon absorption is an open question. DFT calculations of Kr binding energies give, for example, the value of 0.2 eV for Co_8_^+^Kr, higher than the onset at which optical absorption is observed, therefore suggesting that multiple-photon absorption is occurring.

An important observation from the comparison between the experiment in the mid-infrared range and the TD-DFT calculations presented in Fig. 2 is the broadness of the electronic transitions compared to the sharper vibrational bands, as observed also for Au_10_^+^^18^ and Ta_4_^+^^19^. This could be rationalized by the finite experimental temperature (190 K), implying that the observed features involve a large number of rovibronic transitions (Figure S9).

**Fig. 3:**
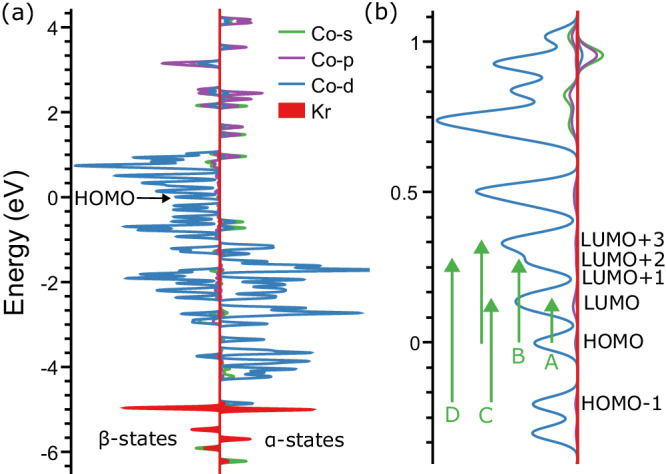
Density of states of Co_8_^+^Kr. **a** DOS of Co_8_^+^Kr, with projections of the states on atomic Kr and Co orbitals. For Co, a distinction is made between *s*, *p* and *d* contributions. **b** Zoom-in around HOMO, depicting the MOs involved in the electronic transitions marked in Fig. 2d.

Given the good correspondence between calculated and measured transitions, a more detailed analysis of the density of states (DOS) will provide insight into their electronic character. Note that the spin magnetic moments of the clusters correspond to those determined by XMCD spectroscopy^14^. Figure 3a presents the DOS of Co_8_^+^Kr, where the *s*, *p*, and *d* contributions are projected onto Co and Kr states. Because of the open-shell nature of the electronic structure of the cluster, the α and β spin states (or spin up and down states, respectively), are plotted separately. It is clearly seen that the low-lying electronic transitions only involve states localized at the cobalt cluster moiety, while the Kr contribution is found at energies much below the HOMO. The DOS around the HOMO is dense, with several *d*-character states of β spin state. Transitions between these states make up the observed absorption spectrum, involving the HOMO and HOMO-1 initial, and LUMO, LUMO + 2 and LUMO + 3 final states, for the transitions labeled in Fig. 2d, and highlighted in Fig. 3b. Hence, the transitions are characterized as intraband in nature, with interband transitions only occurring at much higher excitation energies, above 2 eV. Projecting the initial and final states involved in the transitions onto the MOs of the cluster reveal transitions of essentially single-electron character, except for transition C, involving two initial and two final MOs. From the dense DOS around the HOMO, it is evident that the existence of such low-lying electronic excitations correlates with the high-spin, open *d*-shell, nature of Co_*n*_^+^. We therefore surmise that other high-spin clusters, such as Fe_*n*_^+^ and Ni_*n*_^+^^15^, have similar low-energy excitations.

**Fig. 4:**
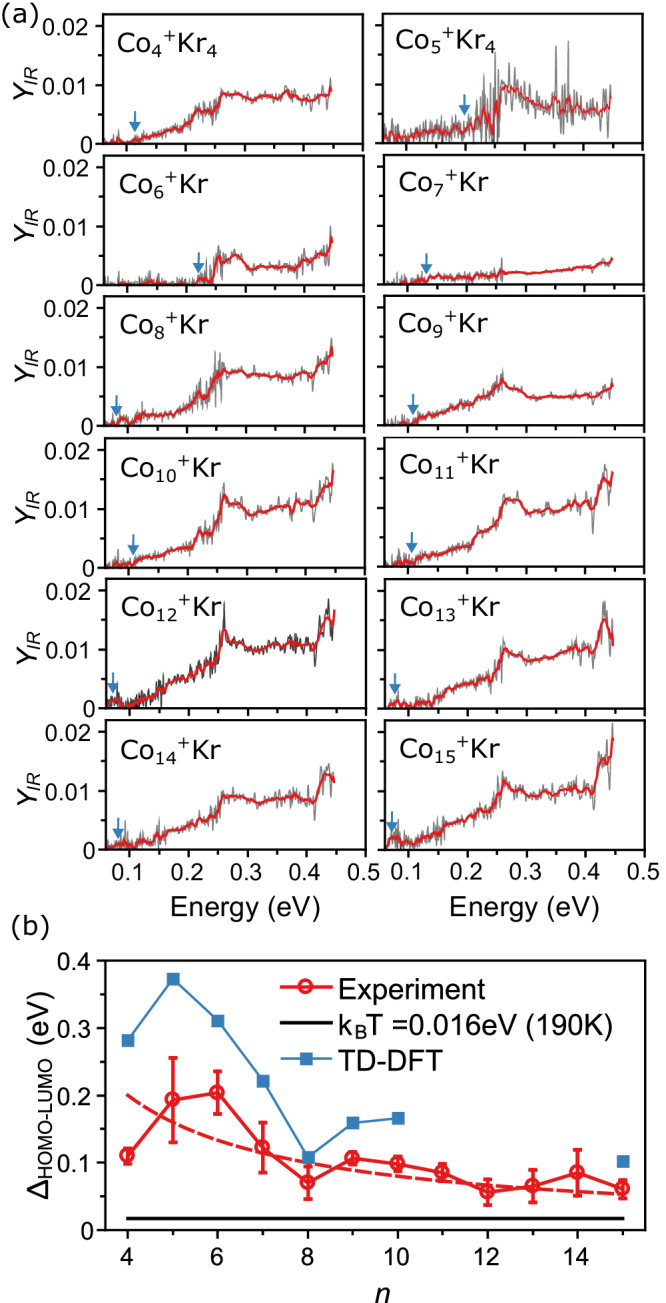
The optical absorption spectra for cationic cobalt clusters. **a** Optical absorption spectra for Co_*n*_^+^Kr_*m*_ (*n* = 4–15), with a 10-point running average (red). Arrows indicate the estimated onsets of optical absorption increase. **b** Optical HOMO-LUMO gaps as a function of *n*, extracted from the experiment (circles) and TD-DFT calculations (squares). The dashed red line is a fit to a *1/n* functional form. Error bars are estimated based on the signal-to-noise ratio of the data for each cluster.

Now that we established the existence of low-lying electronic states in more detail, a natural question that can be addressed is their dependence on cluster size. Figure 4a presents the experimental electronic excitation spectra of cobalt clusters up to Co_15_^+^ (Co_*n*_^+^Kr_*m*_, *n* = 4–15). All clusters show broad optical absorption features attributed to low-lying electronic transitions, while a clear size dependence is observed. For example, Co_6_^+^Kr only starts absorbing light at ~0.25 eV, in contrast to several other species with absorption at photon energies below 0.1 eV. Several clusters show a marked absorption increase at ~0.4 eV, such as Co_8_^+^Kr, while that feature is absent in other species like Co_4,5_^+^Kr_4_. We note that the spectra of most clusters present an abrupt increase in *Y*_IR_ at ~0.26 eV. This corresponds to the frequency above which the third harmonic of FELIX is used, which necessarily has a much lower laser pulse energy *E*_IR_ and for which the optical beam characteristics may deviate from that of the fundamental. Such variations in pulse energy may not be accurately modeled by Eq. (1) and therefore, the feature could be an artifact.

Accurate determination of the onset at which optical absorption starts is difficult to achieve, although it is an important quantity since it relates to the HOMO-LUMO gap of the clusters. Here, we analyze the derivative of the optical absorption curves to infer this onset. The procedure is detailed in Supplementary Note 2 and highlighted in Figs. S10 and S11, with estimated uncertainties based on the experimental noise level.

The resulting experimental HOMO-LUMO gaps are highlighted by arrows in Fig. 4a and are summarized in Fig. 4b. Here it is assumed that the HOMO-LUMO transition has non-zero oscillator strength, which is supported by the TD-DFT calculations. A similar trend is seen in the lowest TD-DFT calculated electronic transitions, also included in Fig. 4b. The agreement between experimental and calculated values is not perfect, but while the computations tend to overestimate the gaps^24^, the trend found by the experiments is correctly reproduced, even replicating the functional form with a minimum for Co_8_^+^Kr.

The TD-DFT calculated low-energy electronic transitions of cationic cobalt clusters were used in the work of Peeters et al. to rationalize the unusually high radiative decay rates of the clusters observed in photofragmentation studies, in combination with statistical modeling of radiative decay rates^10^. The experimentally determined rate constants were fairly well accounted for taking into account the calculated oscillator strengths of the dipole-allowed intraband transitions, an analysis performed up to Co_11_^+^. The experimental confirmation of these transitions by the work reported in this article, thus, is a confirmation of the hypothesis that electronic transitions well below 1 eV are responsible for Recurrent Fluorescence in small cobalt clusters. In fact, using the calculations for Co_15_^+^ performed in the current study, radiation rates can also be computed, further strengthening this point. A comparison of the rates inferred in ref. ^10^ and the values obtained from the TD-DFT calculations, including Co_15_^+^, are presented in Figure S12.

The observed tendency of decreasing HOMO-LUMO gap with size is also interesting in the context of the evolution from discrete well-resolved electron states in small clusters towards the onset of band formation that would coincide with a definition of metallicity of nanoparticles. Historically, this size dependence has been discussed in terms of insulator-to-metal transitions^25^. A material is deemed metallic if the HOMO-LUMO gap is overcome by thermal energy, formulated in the Kubo criterion^26–28^:

2 $${\Delta }_{{{{{{\rm{Kubo}}}}}}}=\frac{4{E}_{{{{{{\rm{F}}}}}}}}{3{N}_{{{{{{\rm{E}}}}}}}}\le {k}_{B}T,$$

with *E*_F_ the Fermi energy, *N*_E_ the number of valence electrons, *k*_*B*_ the Boltzmann constant, and *T* the temperature. The observed decrease with cluster size (up to *n* = 15) is in line with the rough proxy given by Eq. (2). The thermal energy of 0.016 eV relevant here is based on the 190 K source temperature^29^, and is well below the optical HOMO-LUMO gaps deduced from the experiment. Fitting the experimental data to a 1/*n* functional form, however, allows to infer the size *n* at which the clusters show overlapping electronic states at a temperature of 190 K, giving a value *n* = 52 ± 5. Evidently, this estimate does not account for shell structure that can lead to a deviation of the smooth size dependence of Δ_Kubo_.

## Conclusion

In summary, we provide direct experimental evidence for the existence of low-energy transitions in Co_*n*_^+^Kr_*m*_ (*n* = 4–15) clusters, with photon energies below 0.5 eV that are attributed to electronic intraband transitions between states of mainly *d*-character. A wide range of such states exists due to the open *d*-shell electronic structure of the clusters, with consequent high-spin multiplicity. These results confirm the predictions that low-energy electronic states are responsible for fast radiative cooling rates observed for Co_*n*_^+^ clusters^10^. Given the experimental determination of an optical HOMO-LUMO gap, the clusters investigated here are characterized as insulators at the temperature of the experiment (190 K), based on the Kubo criterion. This study shows that the combination of rare gas tagging and broad spectral range IRMPD measurements is a valuable methodology for directly detecting low-energy electronic transitions in gas-phase metal clusters.

## Methods

### Experiment

Positively charged cobalt clusters are produced by laser ablation in a cluster source operating at 20 Hz. A rotating cobalt rod is ablated by the second harmonic of a Nd:YAG laser (532 nm), a few hundred microseconds after a series 9 General Valve injects He gas at a backing pressure of 5 bar. Via collisions with He, the plasma of cobalt atoms is cooled down, triggering cluster production. The source temperature is maintained at 190 K by a constant flow of liquid nitrogen. After expansion into vacuum through a conical nozzle, the formed molecular beam of clusters is collimated by a 2 mm diameter skimmer and a 1 mm diameter planar aperture, before entering a perpendicularly extracted time-of-flight mass spectrometer. To form Kr complexes, 2% of Kr gas is added to the He carrier gas. Infrared spectra are measured by aligning the light of the free-electron laser FELIX counterpropagating with the molecular beam, as shown in Fig. 1a. FELIX is operated at 10 Hz, allowing the consecutive measurement of mass spectra with and without the influence of the infrared light. FELIX is scanned in the 220–3600 cm^−1^ range (0.027–0.446 eV). The fundamental is used from 220 up to 2100 cm^−1^, while its third harmonic is employed from 2100 to 3600 cm^−1^. The laser pulse energy varies with the wavelength, but overall, the pulse energy of the third harmonic is ~10 times lower than that of the fundamental.

### Computational techniques

To interpret the experimental results, DFT and TD-DFT computations are performed for Co_*n*_^+^Kr_*m*_ (*n* = 4, 5 and *m* = 4; *n* = 6–10, 15 and *m* = 1) complexes using ORCA 5.0.3^30^. Based on the work describing the radiative cooling of Co_*n*_^+^^10^, the PBE functional is selected^31^, in combination with the Def2-TZVPP basis set^32^ (for *n* = 4–10). For *n* = 15, the smaller Def2-SVP basis set is used to reduce computational cost. Relativistic effects are considered implicitly by using an all-electron approach and the ZORA approximation, which is crucial for a good agreement with the experimental data. SCF and geometry convergence criteria are set to “Tight”. Geometries for *n* ≤ 8 are taken from previous IR spectroscopic work (Ar messenger)^20^, and spin states from XMCD studies^14,15,33^. For the larger clusters without previous experimental geometrical characterization we rely on our computations.

### Supplementary information

Supporting Information

## Data Availability

The data supporting the findings of this study are deposited in the Radboud Data Repository (10.34973/550s-0062).
