# Supplementary material for: Direct probing of low-energy intra d-band transitions in gas-phase cobalt clusters
Source: Commun Chem. 2024 Jun 4;7:124. doi: 10.1038/s42004-024-01206-2 (PMC11150441; doi:10.1038/s42004-024-01206-2)
Supplement: Supplementary file 1 — Supporting Information [file 42004_2024_1206_MOESM1_ESM.pdf]

Supporting information

Direct probing of low-energy intra *d*-band transitions in gas-phase cobalt clusters

Kevin Anthony Kaw[1], Rick J. Louwerse[2], Joost M. Bakker[2], Peter Lievens[1], Ewald Janssens[1] and Piero Ferrari[1,2,*]

[1]Quantum Solid-State Physics, Departments of Physics and Astronomy, KU Leuven, Celestijnenlaan 200d - box 2414, 3001 Leuven, Belgium

[2]Radboud University, Institute for Molecules and Materials, HFML-FELIX, 6525 ED Nijmegen, Netherlands

*piero.ferrariramirez@ru.nl

Content

1. Comparison of vibrational spectrum of Co_8^+Ar and Co_8^+Kr
2. IR depletion yield spectra of $Co_n^+Kr_m$ clusters
3. Comparison of TD-DFT calculations of Co_8^+Kr with all-electrons and explicit relativistic corrections or with effective core potentials
4. Franck-Condon spectral simulations of vibronic transitions in $Co_4^+Kr_4$
5. Experimental determination of the optical HOMO-LUMO gaps
6. Comparison of measured and computed radiation rates

1. Comparison of vibrational spectrum of Co_8^+Ar and Co_8^+Kr

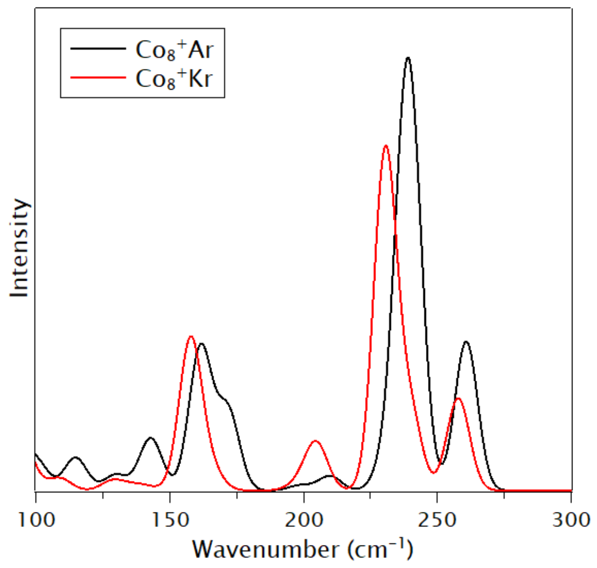

Figure S1. Comparison of computed vibrational spectra of Co_8^+Ar (black) and Co_8^+Kr (red). The same DFT level as described in the main article was selected for both calculations.

2. IR depletion yield spectra of $Co_n^+Kr_m$ clusters

Supplementary Note 1:

In this section we present a comparison of the measured infrared spectra in the far- and mid-infrared spectral ranges, with the calculations of vibrational modes and electronic transitions. Only the sizes with available computations are presented. The TD-DFT calculations presented here are performed with the PBE functional and the Def2-TZVPP basis set, including relativistic effects via the ZORA approximation.

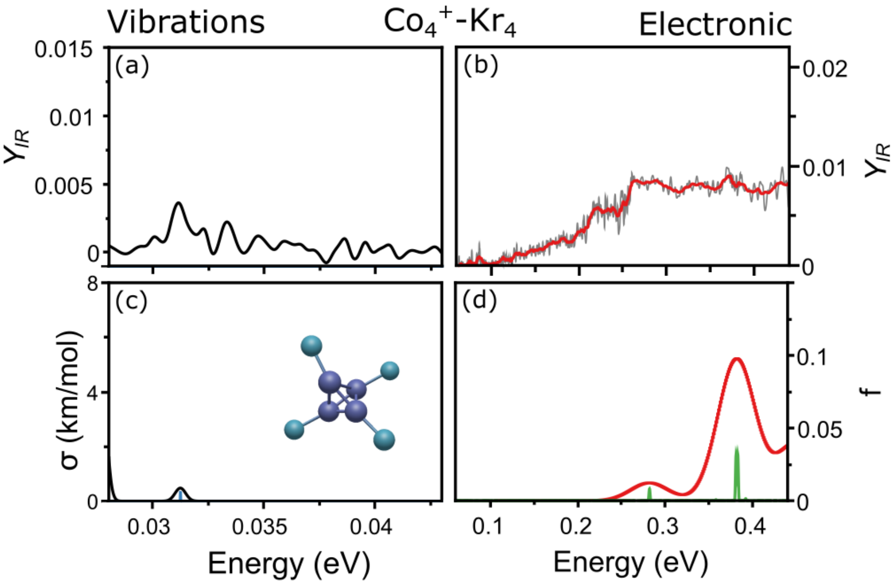

Figure S2. a,b): IR depletion yield spectrum of $Co_4^+Kr_4$. c,d) DFT and TD-DFT calculated spectra.

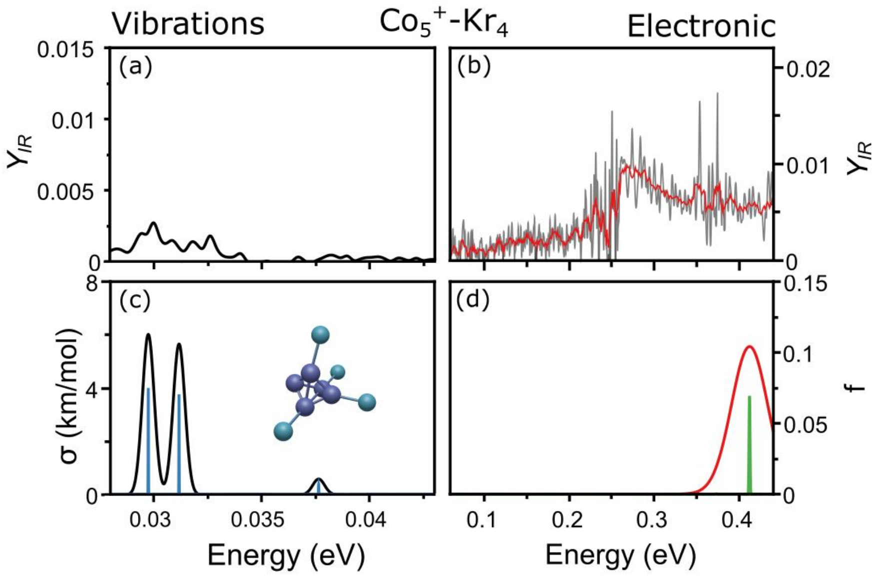

Figure S3. a,b): IR depletion yield spectrum of $Co_5^+Kr_4$. c,d) DFT and TD-DFT calculated spectra.

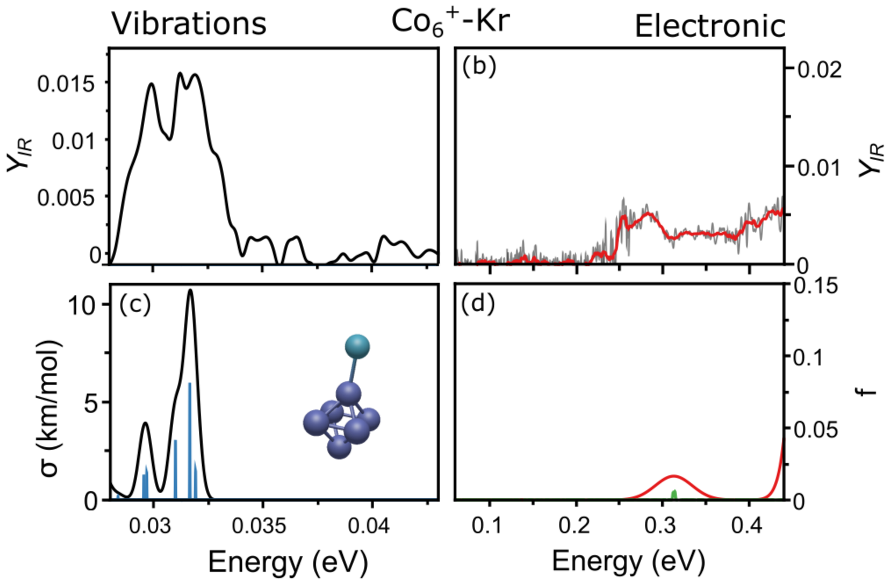

Figure S4. a,b): IR depletion yield spectrum of Co_6^+Kr. c,d) DFT and TD-DFT calculated spectra.

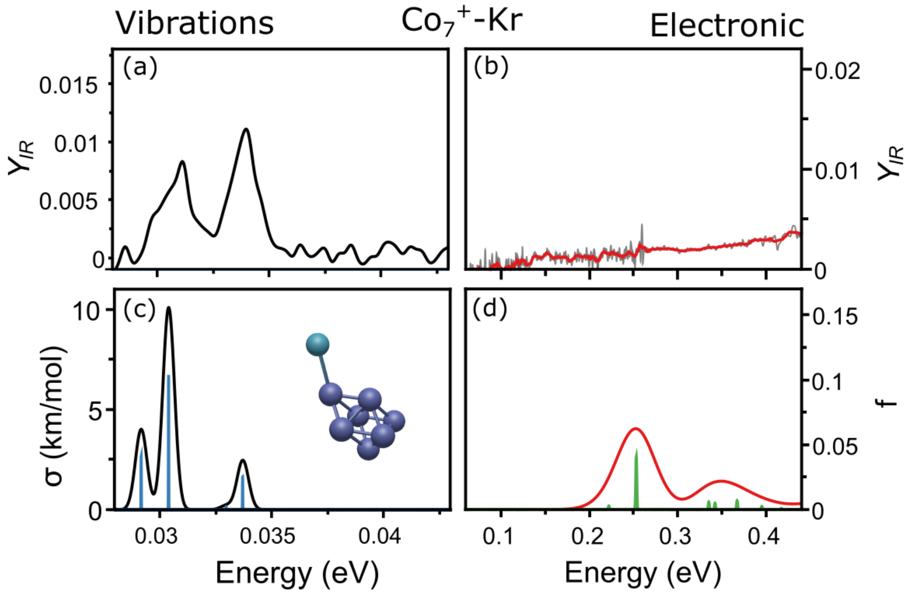

Figure S5. a,b): IR depletion yield spectrum of Co_7^+Kr. c,d) DFT and TD-DFT calculated spectra.

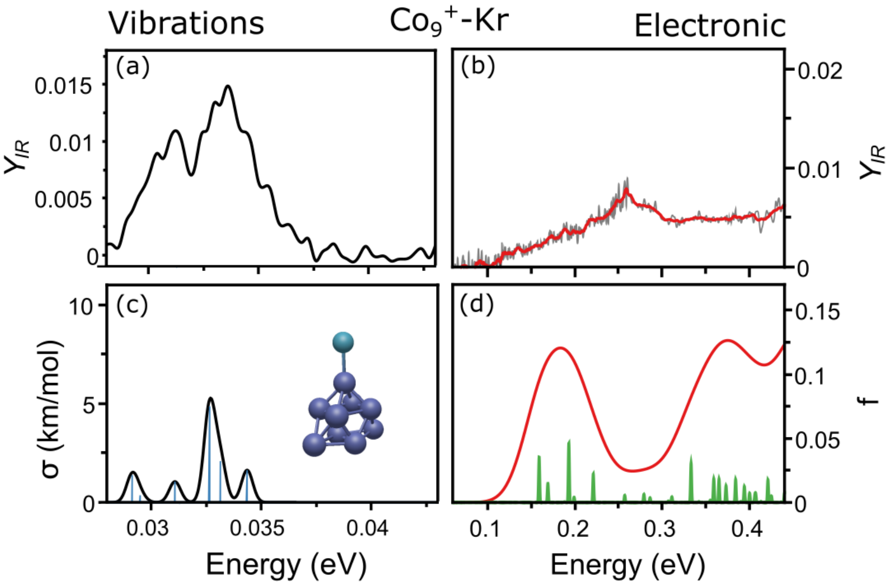

Figure S6. a,b): IR depletion yield spectrum of Co_9^+Kr. c,d) DFT and TD-DFT calculated spectra. While the experimental and computational vibrational modes agree in the wavenumber range, the agreement is not perfect, with the experiment showing broader features. This could be attributed to the high FELIX power used for the measurements, or possibly to the presence of more than one isomer in the molecular beam. Nevertheless, the key observation is the optical absorption observed above 0.1 eV, as predicted by the TD-DFT calculations.

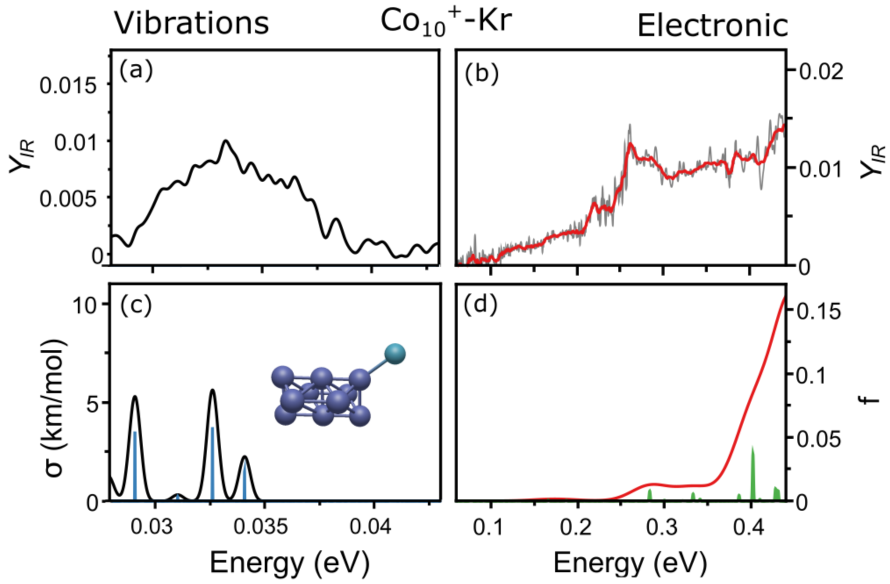

Figure S7. a,b): IR depletion yield spectrum of Co_{10}^+Kr. c,d) DFT and TD-DFT calculated spectra. In this case, the vibrational spectrum is seen much broader than what is predicted by the vibrational calculations. As for the case of Co_9^+Kr, this could be attributed to the high power of FELIX or the presence of multiple isomers. Again, however, the key observation is the optical absorption detected above 0.1 eV, as also predicted by the TD-DFT calculations.

3. Comparison of TD-DFT calculations of Co_8^+Kr with all-electrons and explicit relativistic corrections or with effective core potentials

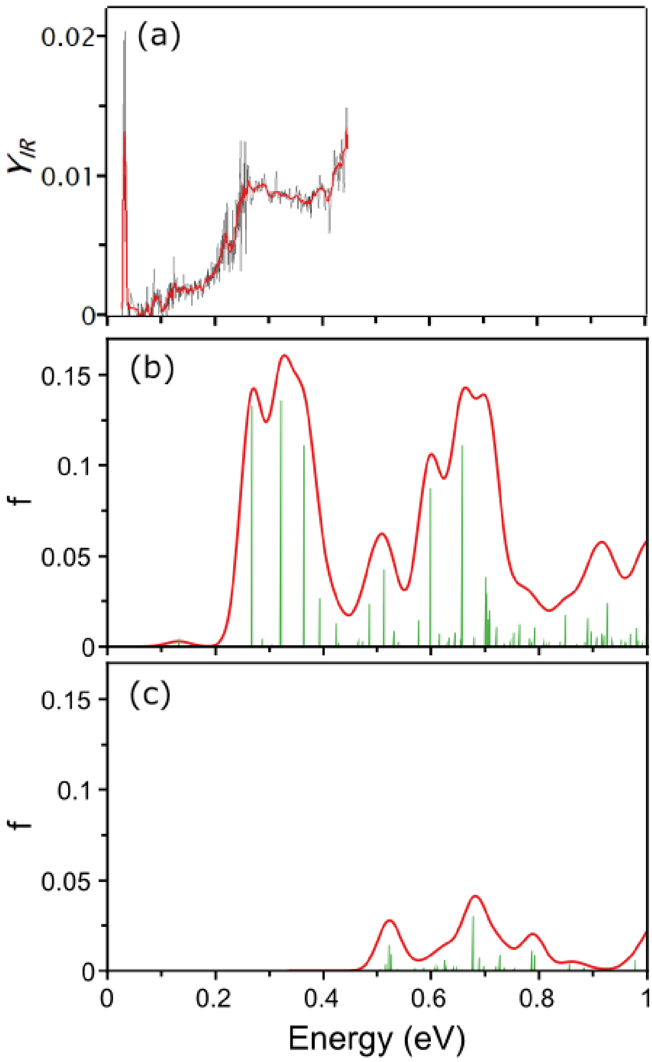

Figure S8. (a) Experimental spectrum of Co_8^+Kr. TD-DFT calculated optical absorption spectrum of Co_8^+Kr using: (b) all electrons with the ZORA relativistic correction, and (c) effective core potentials. In both cases, the same geometry as in the main article is employed, with the PBE+D3/Def2-TZVPP level of theory.

4. Franck-Condon spectral simulations of vibronic transitions in $Co_4^+Kr_4$

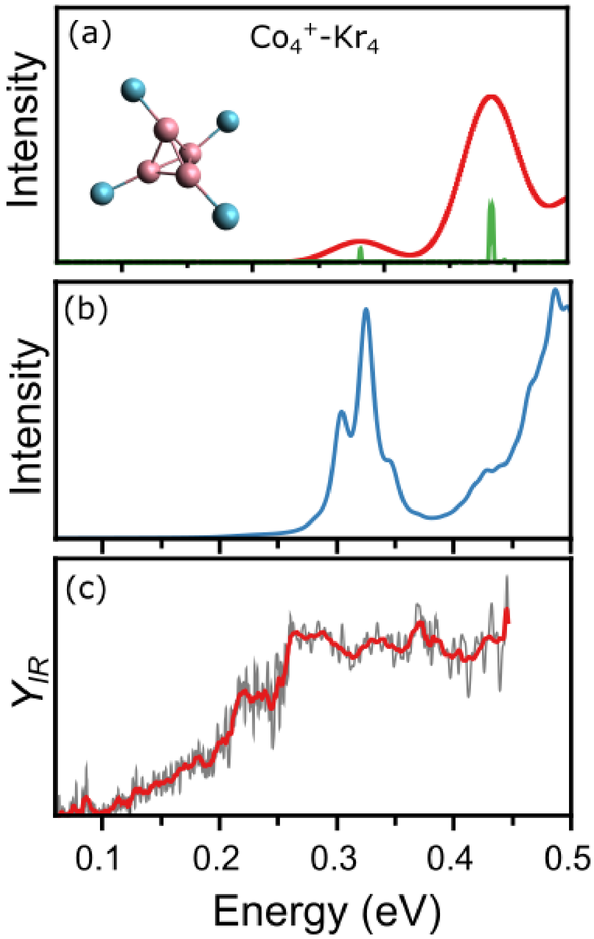

Figure S9. (a) TD-DFT calculation of $Co_4^+Kr_4$. (b) Computed spectrum from the allowed Franck-Condon transitions at a temperature of 190 K. Each transition is broadened by a Gaussian profile with a FWHM of 10 cm^{-1}, corresponding to the spectral line shape of FELIX. The calculation is performed with the Excited State Dynamics (ESD) feature in ORCA 5.03, employing the PBE functional and the Def-TZVP basis set. Effective core potentials (ECP) are employed in this calculation, given the computational cost of the EDS treatment. The Vertical Gradient (VG) model is employed (B. de Souza, G. Farias, F. Neese, R. Izsák, J. Chem. Theory Comput. 2019, 15, 3, 1896–1904), in combination with 10 electronic excited states. (c) Experimental data (same as presented in the main article). While the experimental spectrum is broader than the computation, the agreement between panels (b) and (c) is satisfactory, reproducing a main feature around 0.3 eV and broad absorption features.

5. Experimental determination of the optical HOMO-LUMO gaps

Supplementary Note 2:

In order to estimate the optical HOMO-LUMO gaps from the measured optical absorption spectra, a derivative of the experimental data with respect to the wavenumber was performed. This was preceded by a 10-point running average of the raw data. An increase of the derivative (after the vibration range) is identified as the start of an absorption band. An example is presented in Figure S9, for the case of Co_8^+Kr. In panel (a) the IR depletion yield spectrum of the cluster is presented, corresponding to the data shown in the main article. In panel (b) the derivative of the curve is shown, with the inset zooming in on the region above 400 cm^{-1}. The initial maximum and following minimum below 300 cm^{-1} corresponds to the sharp vibrational modes detected for the cluster in the far-infrared. Above 400 cm^{-1}, instead, changes in the derivative are due to the optical absorption features. The first deviation from zero, at 560 cm^{-1} (0.07 eV), is considered as the optical HOMO-LUMO gap. A summary of the derivatives for the other cluster sizes is presented in Figure S11. We point out that the procedure requires a careful comparison with the corresponding IR absorption spectrum, in order to corroborate that the estimated increase in the derivative correlates with the region where the absorption increase starts. Therefore, the extracted values are treated as an estimation.

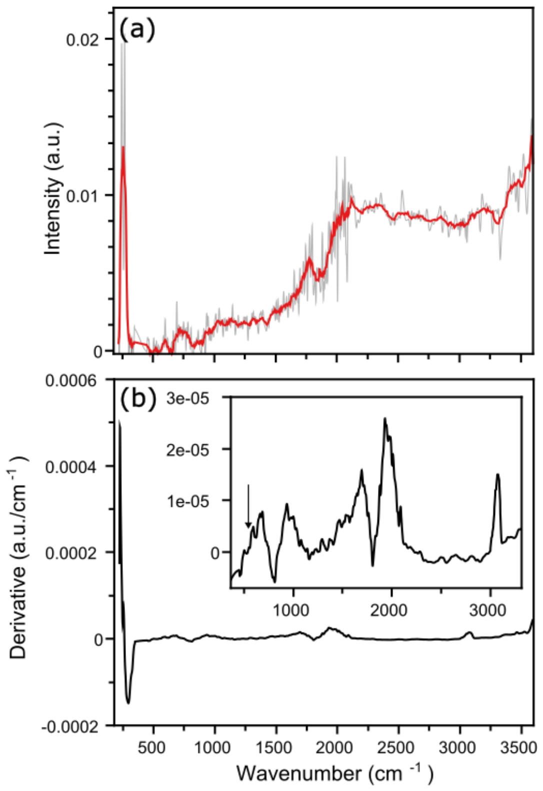

Figure S10. (a) IR depletion yield spectrum of Co_8^+Kr. (b) Derivative of the infrared yield curve of Co_8^+Kr, with the inset showing a zoom in of the region above 400 cm^{-1}. The arrow indicates the deviation from zero.

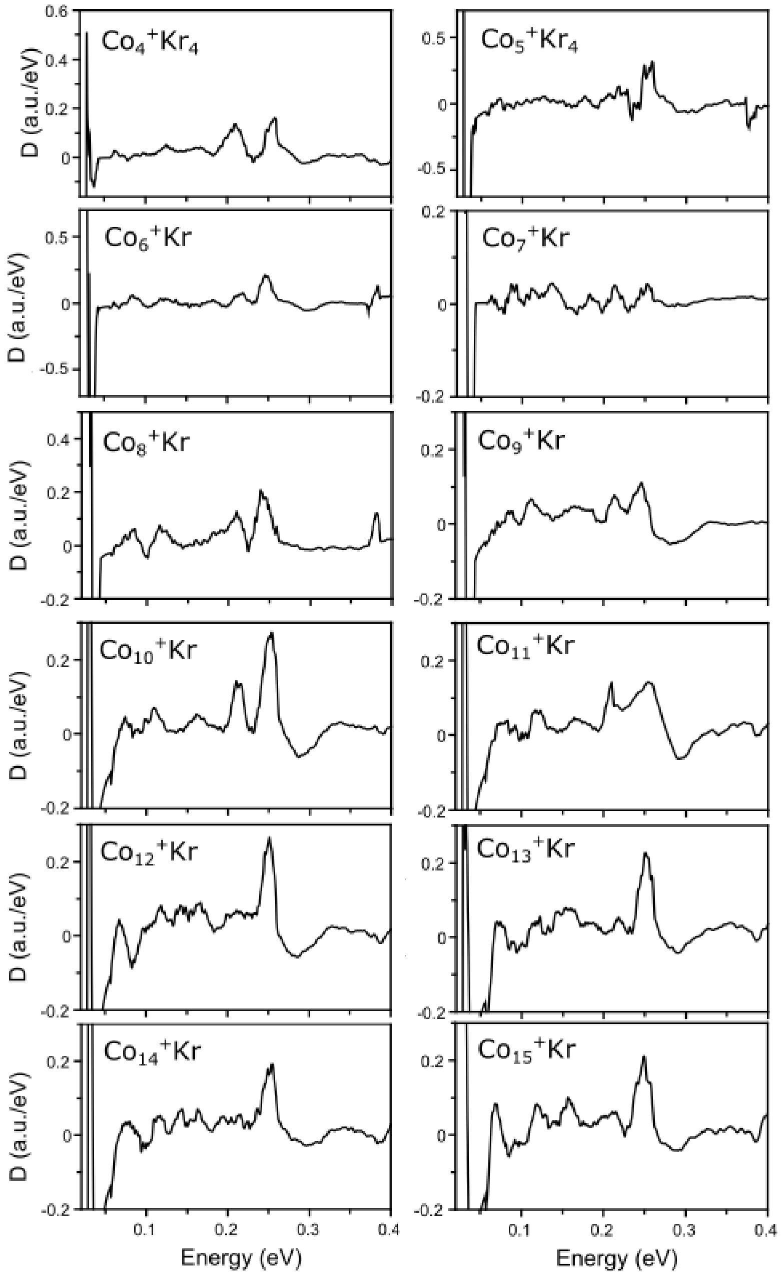

Figure S11. Derivative of the IR depletion yield spectra of $Co_n^+Kr_m$ (n = 4 – 15) clusters.

6. Comparison of measured and computed radiation rates

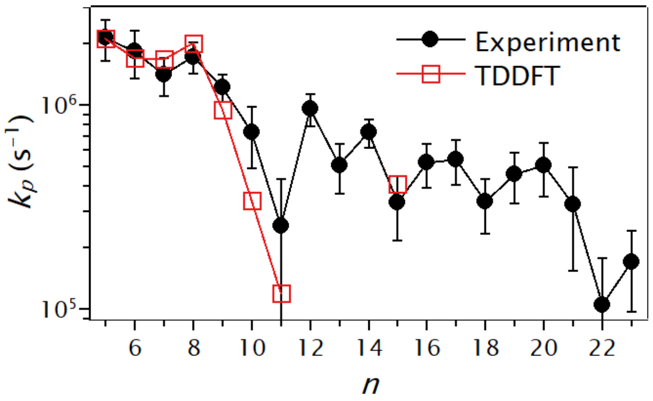

Figure S12. Radiation rates of laser-excited Co_n^+ clusters (black circles), in comparison with rates calculated from the TDDFT calculations. Details in Ref. [10] of main article. The calculations for Co_{15}^+ are included here, using the calculations presented in this work.
